# Antiviral potential of proton-type zeolite

**DOI:** 10.1371/journal.pone.0324484

**Published:** 2025-05-27

**Authors:** Yuka Kimura, Masanori Takemoto, Noriko Nakamura, Takeshi Ichinohe, Makoto Nakakido, Kenta Iyoki, Seiichi Ohta, Nobuhiro Miyamae, Yuki Egami, Koichi Sato, Tatsuya Okubo, Kouhei Tsumoto, Toru Wakihara

**Affiliations:** 1 Department of Chemical System Engineering, The University of Tokyo, Japan; 2 Institute of Engineering Innovation, The University of Tokyo, Japan; 3 Department of Bioengineering, The University of Tokyo, Japan; 4 Department of Infectious Disease Control, International Research Center for Infectious Diseases, The Institute of Medical Science, The University of Tokyo, Japan; 5 Department of Chemistry and Biotechnology, The University of Tokyo, Japan; 6 Laboratory of Medical Proteomics, The Institute of Medical Science, The University of Tokyo, Japan; 7 Nippon Paint Co., Ltd, Japan; Beijing University of Chemical Technology, CHINA

## Abstract

This study reports the potent antiviral ability of proton-type zeolites without the aid of any metal cations. Antiviral activities of zeolites with different topologies and chemical compositions are investigated using M13 phage and influenza virus. Proton-type zeolites exhibit excellent antiviral activity, equivalent to 99% inactivated. Antiviral tests using a centricon device suggest that direct contact of viruses on the external surface of zeolites is required to inactivate the viruses. This discovery is of importance in an interdisciplinary research field covering both zeolite science and virological science and shed light on the possibility of the development of low-cost and environmentally friendly antiviral zeolites.

## Introduction

Given the threat of COVID-19, the importance of antiviral research has been well understood in recent years [1–3]. Since viral infection can rapidly and easily spread in today’s global society with the development of transport network, multiple-barrier protection is required to avoid disease outbreaks [4]. In particular, contact infection is a representative cause of infection in public spaces [5]. Designing antiviral surfaces has drawn researchers’ interest in decreasing the risk of infection [6,7].

Zeolites are a family of crystalline, microporous materials with 3-dimentional channel systems. Partial substitution of tetrahedral Si by tetrahedral Al imparts acidity and cation exchange ability to zeolites, allowing to be practically utilized in diverse applications such as detergent builders [8] and gas purifications [9]. In terms of antibacterial and antivirus application, cation-exchange ability has been recently focused on owing to their potential as host materials to accommodate metal species, such as Ag and Cu, well known to be active against bacteria [10–12] and virus [13,14]. Ag-zeolite for an antimicrobial agent has been actually commercialized in 1984 [15]. Metal exchanged zeolites certainly exhibit antibacterial and antivirus activities with the aid of such metal species, whereas such strategy is said to still have not only the problem of cost increase, but also bacterial resistance to silver, [16] color change of products, [17] formation of insoluble precipitates (e.g., AgCl or Ag_2_S) [18] and toxicity of silver species [19]. Therefore, further suitable material design is highly required to realize the practical application of zeolites without suffering from above-mentioned drawbacks.

This contribution represents antivirus activities by proton-type (H-type) zeolites without the aid of metal species. Antivirus activities of zeolites with different topologies and chemical compositions were evaluated by a high-throughput method. [20] Antiviral tests using M13 phage as a model virus revealed that H-type zeolites inactivated M13 phage as well as metal-exchanged zeolites. Furthermore, high antiviral ability of H-type zeolites was also observed in the case of influenza virus strain A/PR8 (PR8) as another model virus. Antiviral tests using a centricon device indicated that direct contact of viruses on the external surface of H-type zeolites was required to inactivate the viruses. H-type zeolites have been the focus of industrial attention as solid acid catalysts so far [21], but the acid properties of zeolites allow them to be potentially as antiviral materials. Antiviral features of H-type zeolites for both non-enveloped and non-enveloped viruses would play a significant role in designing environmentally friendly materials showing antiviral activity without the aid of metal cations.

## Experimental

### Materials

Potassium hydroxide (KOH, 85%), aluminum hydroxide (Al(OH)_3_, 95%), sodium chloride (NaCl, 99.5 + %), sodium nitrate (NaNO_3_, 99.0 + %), copper nitrate trihydrate (Cu(NO_3_)_2_·6H_2_O, 99.0-104.0%), hydrochloric acid (HCl, 35 wt%), hydroﬂuoric acid (HF, 46–48 wt%), D(+)-Glucose (98.0 + %), Agar (Powder), ampicillin, kanamycin, |phosphate-buffered saline (1 × PBS(−), 164–25511) were purchased from FUJIFILM Wako Pure Chemical Corporation. Boric acid (H_3_BO_3_, 99.99%) was purchased from Thermo Scientific Chemicals. 4-(2-hydroxyethyl)-1-piperazineethanesulfonic acid (HEPES), Yeast Extract Dried (15838–45) and tryptone (35640–95) were purchased from NACALAI TESQUE INC. *Escherichia coli* (*E. coli*) strain XL-1 Blue suspension was supplied from Agilent technologies. Polyethylene glycol (PEG 6000) was purchased from Merck KGaA. Sodium hydrogen carbonate (NaHCO_3_), LUDOX® HS-40 and trypsin acetylated from bovine pancreas (T6763) were purchased from Sigma-Aldrich. Commercially available LTA zeolite (Zeoal 4A) was purchased from Nakamura Choukou Co., Ltd. Commercially available FAU type zeolites, such as HSZ-320NAA, HSZ-320HOA, HSZ-350HUA and HSZ-390HUA, were purchased from Tosoh Corporation.

### Synthesis of EDI zeolite

According to a previous paper, [22] EDI zeolite was synthesized as following procedure. In a plastic bottle, KOH and Al(OH)_3_ were mixed in distilled water, and kept stirring at 400 rpm while heating using a hot stirrer set at 100 °C overnight to yield a clear solution of KAlO_2_. In another plastic bottle, KOH and LUDOX® HS-40 were mixed in distilled water to yield a solution of K_2_SiO_3_. After lowering the temperature of both solutions to room temperature (20–25 °C), the solution of KAlO_2_ was slowly added into the solution of K_2_SiO_3_ under stirring at 400–500 rpm. After the dropwise addition of the solution KAlO_2_, the obtained gel was stirred manually with a spatula to yield slurry. The molar composition in the reactant was set at 1.5SiO_2_: 1Al_2_O_3_: 12K_2_O: 61H_2_O. The slurry was heated at 100 °C for 3 h, and then the solid product was collected by the centrifugation at 14000 rpm for 2 min (Model: 3740, Rotor: AF-5004CH, KUBOTA Crop.) and washed with water until pH = 7. The solid product was dried at room temperature (20–25 °C) under vacuum (<−0.08 MPaG) overnight.

### Preparation of cation exchanged zeolites

Ion exchange was carried out by mixing zeolite powder and metal nitrate solution (0.01 M), wherein a solid to liquid ratio was set at 1: 20 by weight. The mixture was stirred at 500 rpm for 2 h at room temperature (20–25 °C). The solid product was collected by centrifugation at 14000 rpm for 2 min, washed with distilled water over two times and dried at room temperature (20–25 °C) under vacuum (<−0.08 MPaG) overnight. In the case of EDI zeolite, prior to Ag exchange, Na exchange was also carried out for as-synthesized EDI zeolite because as-synthesized EDI zeolite was obtained in a K form. The procedure was the same except for the use of the solution of NaNO_3_ (0.1 M).

## Characterization

Powder X-ray diffraction (PXRD) patterns were collected by using an X-ray diffractometer (Smart Lab, Rigaku) with CuKα radiation (λ = 0.15406 nm) at 45 kV and 200 mA over an angular range of *2θ* = 3–50° with a scanning step of 0.02° at a scanning speed of 4°/min. Transmission electron microscopy (TEM) images were obtained from JEM-1400 (JEOL). Chemical compositions of the zeolite samples were determined by inductively coupled plasma atomic emission spectroscopy (ICP-AES) using ICPE-9820 (SHIMAZU Corp.). Approximately 6 mg of zeolite sample was added to a 50 mL plastic bottle, followed by the addition of 2 mL of ultrapure water, 1 mL of HCl solution, and 0.5 mL of HF solution. This bottle was sealed and left to stand for 1 h at room temperature (20–25 °C) to completely dissolve the solid product, followed by the addition of 12.5 mL of the solution of H_3_BO_3_ (4 wt%) for complexing the excess HF in the solution. Ultrapure water was then added to the bottle until the total volume of the solution reached 50 mL. This solution was further diluted 5 times with ultrapure water before the ICP-AES measurement. For the elemental analysis of the liquid phase, approximately 5 μL of the liquid product was added to 25 mL of ultrapure water, and then this solution was directly used for ICP-AES measurement without further treatment. The acidity of the samples was examined using temperature-programmed desorption of NH_3_ (NH_3_-TPD). NH_3_-TPD profiles were recorded on a BELCAT II instrument (MicrotracBEL Corp.) using a thermal conductivity detector (TCD). 50 mg of the samples were pretreated at 500 °C for 1 h in He (50 sccm), and absorbed 5.19% NH_3_/He (50 sccm) at 100 °C for 30 min. He was passed through the reactor for another 15 min. Desorption was carried out under He flow (30 sccm) by increasing the temperature up to 650 °C at a rate of 10 °C/min.

### Antiviral test

#### Preparation of M13 bacteriophages.

Phage stocks were prepared as described previously [23]. NaCl (2.5 g), tryptone (8.0 g), and yeast extra (5.0 g) were dispersed in MilliQ-water (475 mL) and the mixture was autoclaved at 120 °C for 20 min to yield a TY medium. 25 mL of filtered 20% glucose solution and 1.0 mL of ampicillin solution with a concentration of 50 mg/mL were added to the TY medium. *E. coli* stock containing an antibody library was grown in prepared broth in the shaker at 37 °C, 180 rpm until OD_600_ = 0.4. The prepared *E. coli* was infected with helper phages (10 mL of 2 × 10^12^ cfu helper phage to 500 mL broth) at 37 °C for 30 ~ 60 min in water bath. The culture was centrifuged at 3200 G for 10 min at 4 °C, and the pellet was resuspended in 500 mL of a TY medium, which was prepared by mixing NaCl (2.5 g), tryptone (8.0 g), yeast extra (5.0 g), MilliQ-water (475 mL), filtered 20% glucose solution (2.5 mL) ampicillin with a concentration of 50 mg/mL (1 mL) and kanamycin with a concentration of 50 mg/mL (0.5 mL) and was autoclaved at 120 °C for 20 min. The *E. coli* infected with helper phages was grown overnight at 25 °C while stirring at 190 rpm. The culture was centrifuged at 7000 G for 10 min at 4 °C and the supernatant was recovered. 250 mL of the supernatant was mixed with 63 mL of PEG solution containing NaCl (14.9 wt% PEG 6000 and 10.8 wt% NaCl), which was filtered with 0.2 µm filter prior to mixing, and the mixture was incubated on ice bath for 1 h. The supernatant with PEG solution was centrifuged at 12000 G for 30 min at 4 °C. The pellet was resuspended in 20 mL of PBS buffer and centrifuged again at 12000 G for 30 min at 4 °C. 5 mL of PEG solution was added to the resuspended pellet and the mixture was incubated on ice for 10 min, and then centrifuged at 12000 G for 30 min, at 4 °C. The pellet was dispersed in SM buffer (100 mM NaCl, 50 mM Tris-HCl, 8 mM MgSO_4_/7H_2_O) and stored at 4 °C.

#### Antiviral test against M13 bacteriophage.

Antivirus activity of zeolites was compared by a high-throughput method reported in a previous study [20]. Prior to antivirus tests, TYE plates were prepared as follows. Agar (7.5 g), NaCl (2.5 g), tryptone (4.0 g) and yeast extract (2.5 g) were mixed in milliQ-water (475 mL) and autoclaved at 120 °C for 20 min. Following the sterilization by autoclave and cooled down to 60–70 °C, filtered 20% glucose and ampicillin with a concentration of 50 mg/mL were added to the autoclaved solution. The product was poured with the liquid level of about 5 mm to the plastic plate. After the agar got coagulated, the plates were covered, wrapped with plastic wrap or plastic bag to avoid getting dried, and then stored at fridge upside down to avoid the condensation.

10 mg of each sample and 90 μL of the diluted M13 phage suspension in PBS buffer were prepared in the 1.5 mL tubes and incubated overnight at room temperature (20–25 °C) on the rotating mixer. After overnight incubation, the samples were centrifuged at 12000 G for 2 min (MiniSpin^®^ plus, eppendorf). 10 µL of M13 were collected from each sample and made a series of 1/10 dilutions. The prepared serial-dilution of M13 phages were added to 90 µL of *E. coli* strain XL-1 Blue suspension at OD_600_ = 0.4 ~ 0.6 and incubated in water bath at 37 °C for 30 min. 5 μL of *E. coli* infected with each M13 dilution were spotted onto the grids on the TYE plates, which was repeated 3 times for each sample. After incubation at 37 °C overnight, the emerged colonies in the grids were counted and the number of active phages were calculated.

Antivirus test against M13 phage using centricon filter was also carried out as follows: Amicon Ultra 0.5 mL tube (NMWL: 3K, Merck) were used as a test container. 1100 µL of 20 mM HEPES buffer and 110 mg of zeolite samples were placed prepared in the outer side of the filter, while 150 µL of M13 suspension was sealed in the inner side. The tubes were tightly closed, well-mixed by shaking and incubated for 48 h at room temperature (20–25 °C). After the incubation, 10 µL of M13 suspensions were collected from each sample and made a series of 1/10 dilutions. The prepared serial-dilution of M13 phages were mixed with *E. coli* strain XL-1 Blue cells at OD_600_ = 0.4 ~ 0.6 and incubated in water bath at 37 °C for 30 min. After the incubation, 5 μL of *E. coli* infected with each M13 dilution were spotted onto the grids on TYE plates, which was repeated 3 times for each sample. After overnight incubation at 37 °C, the emerged colonies in the grids were counted and the number of active phages were calculated.

#### Antiviral test against influenza virus PR8.

Madin-Darby canine kidney (MDCK) cells were maintained in minimal essential medium (MEM) (Nacalai Tesque, 21443–15) supplemented with 10% v/v fetal bovine serum (FBS) and 1% v/v penicillin (100 units/mL)/streptomycin (100 μg/mL). The influenza virus A/PR8 was grown in allantoic cavities of 10-fold fertile chicken egg for 2 days at 35 °C. The stock virus titer was quantified by a standard plaque assay using the MDCK cells and stored at −80 °C [24] 100 mg (or 10 mg) of zeolites and 900 µL of 20 mM HEPES buffer were added in the 1.5 mL tubes. 100 µL of PR8 virus suspension containing 5 × 10^7^ plaque forming unit (pfu) was added to each tube. For the blank, 900 µL of 20 mM HEPES and 100 µL of PR8 virus suspension was mixed. The samples were incubated for 24 h at room temperature (20–25 °C) on a rotating mixer. After the 24 h incubation, the samples were centrifuged at 10000 rpm for 5 min at 4 °C (MDX-310, TOMY). Virus titers were determined by inoculation of MDCK cells in six-well plates with serial tenfold dilution of the samples (200 µL) at 37°C. After 1 h of incubation, cells were washed thoroughly with PBS and overlaid with 2 mL agar medium. The number of plaques in each well was counted 2 days after incubation. This experiment was repeated 3 times for each sample and two biological replicates were generated.

## Results and discussion

**Fig 1:**
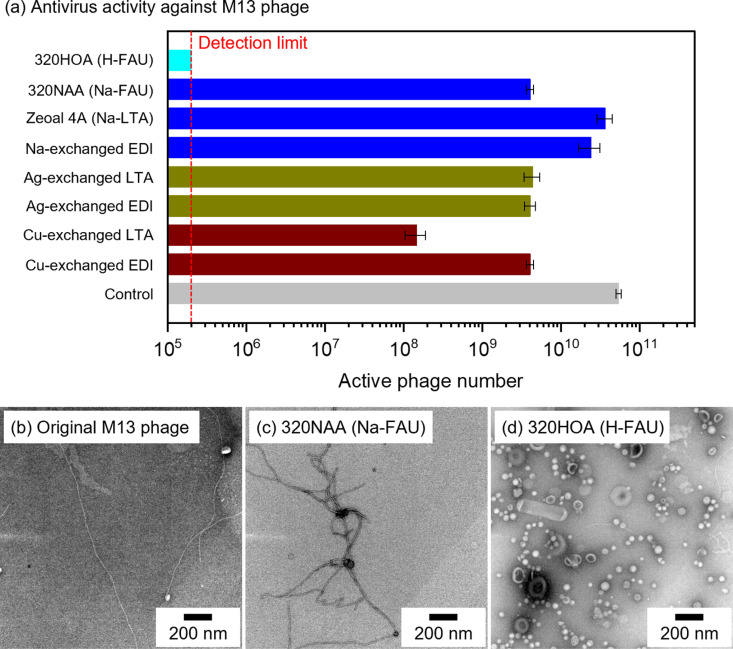
(a) Antivirus activity of zeolites against M13 phage. (b-d) TEM images of the original M13 phage and counterparts after the antivirus tests using 320NAA and 320HOA.

Table 1 summarizes zeolite samples and their chemical compositions. Briefly, PXRD patterns of parent and cation-exchanged zeolites confirmed that crystalline structures of the original zeolites retained after cation exchange (S1 Figs–4). Antivirus activities of different zeolites against M13 phage, a filamentous bacteriophage widely utilized for phage display technology, were compared in Fig 1(a), which showed the active M13 phage titer together with the control experiment. Decrease of active M13 phage titer were observed in Ag-exchanged (shown as Ag-exchanged LTA and Ag-exchanged EDI) and Cu-exchanged (shown as Cu-exchanged LTA and Cu-exchanged EDI) zeolites. Compared to Ag-exchanged and Cu-exchanged zeolites, Na-type zeolites (shown as FAU, LTA and EDI) exhibited poor activities. However, in the case of H-type zeolite (shown as 320HOA (H-FAU)), the active M13 phage titer was below detection limit, which indicated that more than 99% of M13 phage were inactivated by 320HOA despite of the absence of metal cations. Figs 1(b-d) shows TEM images of the original M13 phage and counterparts after the antivirus experiments using 320NAA and 320HOA zeolites. The original M13 phage has a filamentous morphology (Fig 1(b)). The filamentous morphology retained in Fig 1(c), whilst the portion of spherical moieties drastically increased in the case of 320HOA (Fig 1(d)). Previous papers have reported that filamentous phages like M13 phage turned to be spherical after inactivation.[25,26] Therefore, the increase of spherical moieties observed in Fig 1(d) further supported that 320HOA has a potential to inactivate virus without the aid of metal species such as Ag and Cu.

**Table 1:** List and chemical compositions of zeolite samples.

No.	Sample Name	Product Name	Topology	Cation	Si/Al	M/Al (M = Ag or Cu)
1	Zeoal 4A (Na-LTA)	Zeoal 4A	LTA	Na^+^	1.3	–
2	Ag-exchanged LTA	–	LTA	Ag^+^	0.9	0.03
3	Cu-exchanged LTA	–	LTA	Cu^2+^	1.0	0.04
4	Na-exchanged EDI	–	EDI	Na^ + ^, K^+^	1.0	–
5	Ag-exchanged EDI	–	EDI	Ag^+^	1.0	0.04
6	Cu-exchanged EDI	–	EDI	Cu^2+^	1.0	0.04
7	320HOA (H-FAU)	HSZ-320HOA	FAU	H^+^	2.8	–
8	320NAA (Na-FAU)	HSZ-320NAA	FAU	Na^+^	2.8	–
9	350HUA (H-FAU)	HSZ-350HUA	FAU	H^+^	5.0	–
10	390HUA (H-FAU)	HSZ-390HUA	FAU	H^+^	250	–

**Fig 2:**
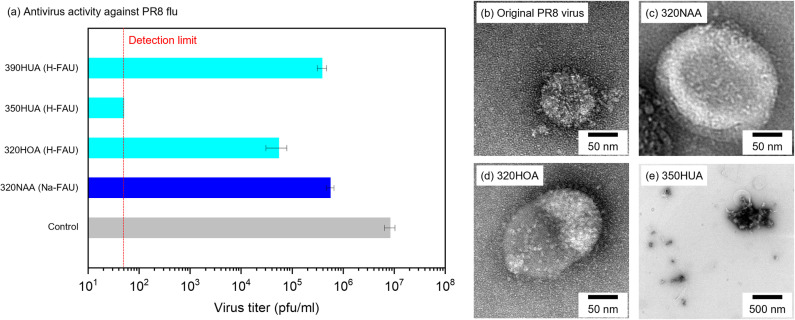
(a) Antivirus activity of zeolites against PR8. (b-e) TEM images of the original PR8 and counterparts after the antivirus tests using 320NAA, 320HOA and 350HUA.

Antivirus activities of commercially available zeolites against influenza virus PR8 were also compared in Fig 2(a), wherein 390HUA (H-FAU), 350HUA (H-FAU), 320HOA (H-FAU) and 320NAA (Na-FAU) were used as received. The virus titers in all tested zeolites were below that in the control experiment, which indicated that H-type and Na-type zeolites have antivirus activity in the absence of metal species. In particular, 350HUA exhibited the highest antivirus activity among tested zeolites, enough to inactivate PR8 virus to below detection limit. Figs 2(b-e) shows TEM images of the original PR8 virus and ones after the antivirus tests using 320NAA, 320HOA and 350HOA. PR8 virus showed spike proteins protruding from the spherical core (Fig 2(b)). PR8 virus inactivated by 320NAA and 320HOA were partially chipped from its original structure (Figs 2(c-d)). As for 350HUA, showing the highest antivirus activity against PR8 virus among the tested zeolites, few viruses with spherical morphologies of PR8 virus were observed and it appeared to have completely collapsed (Fig 2(e)). These results were consistent with antivirus activities as mentioned above. It is further confirmed that H-type zeolites have potent antivirus properties in the absence of metal cations. In the case of Ag-exchanged zeolites, it should be widely accepted that released Ag ion plays the important role in antibacterial and antivirus applications. On the other hand, H-type zeolites have no active components showing such functionality. As far as ability for protein denaturation was investigated using an enzyme-linked immunosorbent assay (ELISA), no protein denaturation by proton-type zeolites was observed (S5 Fig). Therefore, antiviral mechanism by H-type zeolites is still unclear.

**Fig 3:**
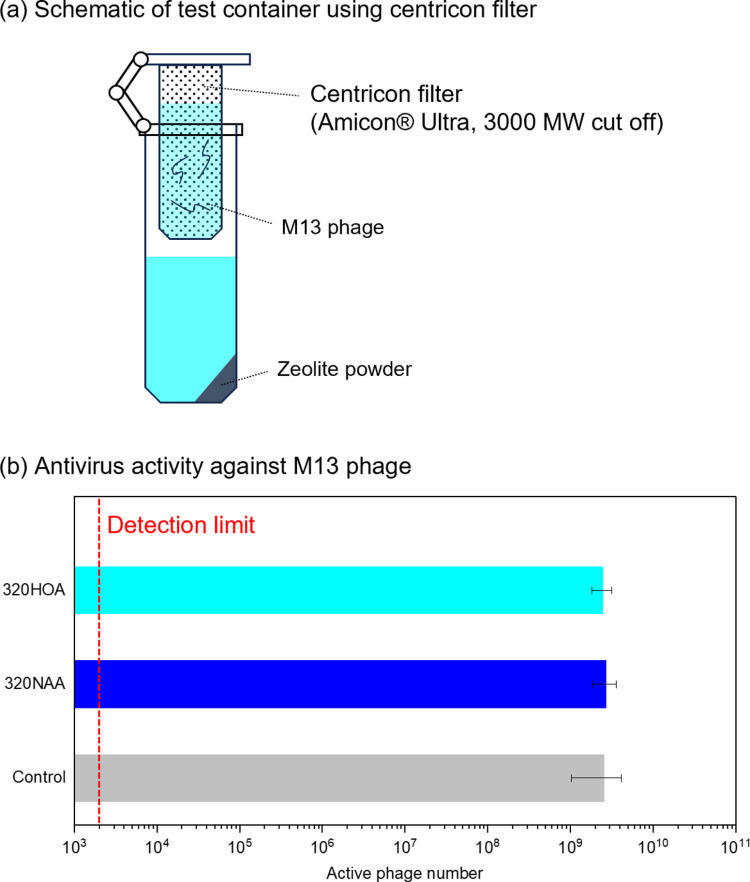
(a) Schematic of test container using centricon filter (Amicon, 3000 MW cut off). (b) Antivirus activity of zeolites against M13 phage.

To understand the origin of their antiviral activity, we performed the antivirus experiment using centricon filter (Amicon, 3000 MW cut off), by which M13 phage could not directly contact with zeolite during the antivirus test (Fig 3(a)). Fig 3(b) shows antivirus activity of zeolites against M13 phage. In contrast to the experiment without cetricon filter shown in Fig 1(a), the significant decrease of active M13 phage were not observed. This result implied that direct contact of M13 phage to zeolites was mandatory to inactivate virus by H-type zeolites.

**Fig 4:**
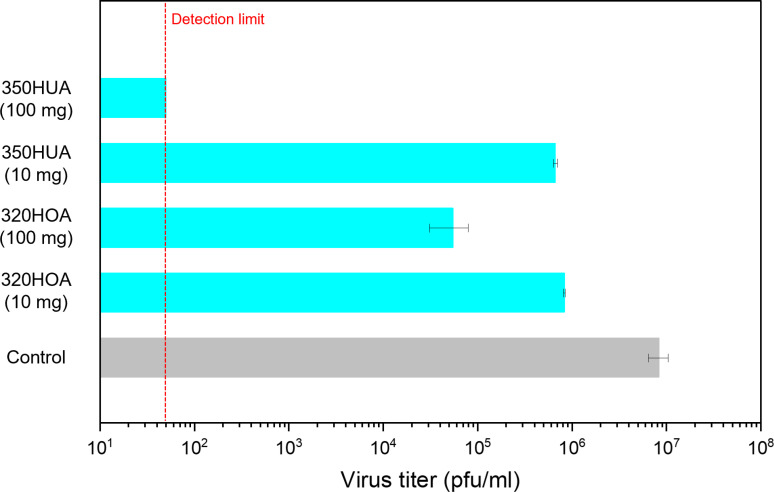
Effect of sample dose for antivirus activity against PR8 virus.

Acidity is one of the most important characteristics of zeolite which allows it to be utilized in acid catalysis [21]. Partial substitution of Si atoms in 3-dimentional SiO_2_ framework by Al atoms and negative charge of aluminosilicate framework is compensated by cations (e. g., H^+^, Na^+^, K^+^), resulting in the formation of Brønsted acid sites. Acid-catalyzed reactions over aluminosilicate zeolites, such as cracking and isomerization, reactions occur on the external micropore (external surface) and/or internal micropore space. Considering the sizes of M13 phage and PR8 virus, these viruses could not penetrate into micropore spaces. Therefore, it can be assumed that antivirus activity of H-type zeolites is originated from acid sites on the external surface of zeolite particles. Actually, antivirus activity of zeolites decreased with the decrease in sample dose (Fig 4). This implies that decreased frequency of contact between viruses and zeolite particles results in the decrease of antivirus activity.

**Fig 5:**
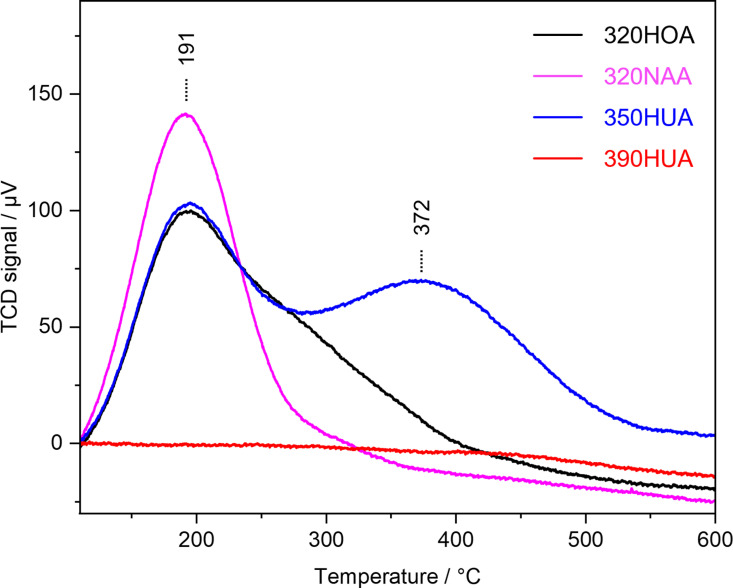
NH_3_-TPD profiles of zeolite samples used in the anti-virus activity test for PR8 virus.

**Fig 6:**
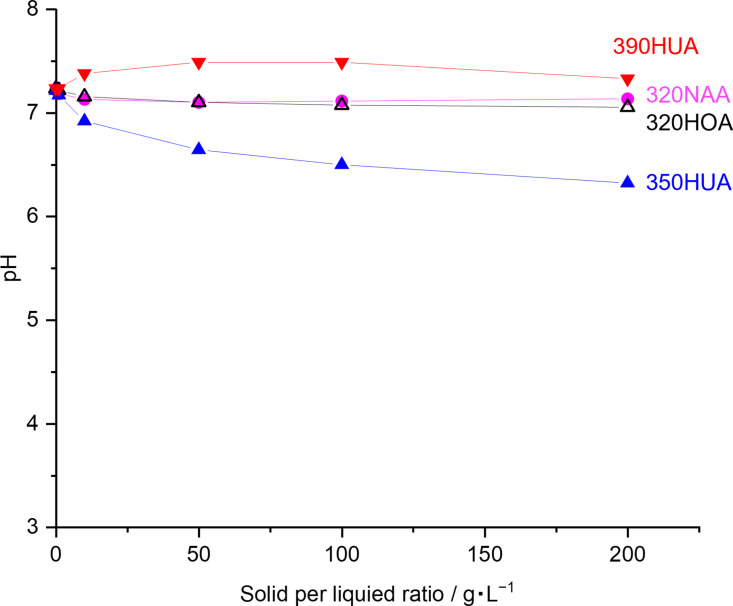
pH of the reaction solutions containing different amounts of zeolites powder. 20 mL of 20 mM HEPES buffer was prepared in the centrifuge tube and followed by the addition of different amounts of zeolite. Before the measurement, each slurry was well-mixed by VORTEX mixer (Vortex-Genie 2, Electro Scientific Industries, Inc.).

All tested aluminosilicate zeolites used in this contribution exhibit acid properties at least. Acid properties of zeolites depend on their topologies, chemical compositions and exchanged cations. Fig 5 shows NH_3_-TPD profiles of zeolite samples used in the anti-virus activity test for PR8 virus. NH_3_-TPD profile of 390HUA has no peak due to its low Al content (Si/Al = 250). Desorption peak at 191 °C was detected in NH_3_-TPD profiles of 320NAA, 320HOA and 350HUA. This peak is assigned to weak acid sites present in zeolite. Furthermore, a peak at 372 °C was also observed in the case of 350HUA, which confirmed that 350HUA possessed stronger acid sites compared to other zeolites. Comparing 320HOA and 320NAA, where chemical composition is the same except for compensate cations, 320HOA exhibited a stronger acid strength and possessed a larger amount of acid sites. This result is consistent with the antivirus activity demonstrated in Fig 2(a). Although 390HUA exhibited an antivirus activity shown in Fig 2(a), its antivirus activity was poorer than 350HUA and 320HOA. This is probably because the amount of proton compensating the negative charge of Al in 390HUA (Si/Al = 250) is quite low compared to others (Si/Al ratios of 350HUA and 320HOA are 5.0 and 2.5, respectively.) There is a possibility that pH changes of the test solutions by the addition of zeolites with acidic properties leads to inactivation owing to pH-sensitive structures of virus (*e.g.*, M2 ion channel in influenza virus is activated at acidic environments [27]). However, pH of the solution was near neutral (Fig 6). This further suggested that direct contact would be the initial step to inactivate virus by H-type zeolites. In terms of practical applications, zeolite should be required to be molded with binders into appropriate shapes (e.g., pellet, film, beads). Therefore, it is also necessary to design meso/macro structures of the molded products containing zeolite moieties to facilitate contact between proton-type zeolites and viruses.

## Conclusion

This contribution first demonstrated the antivirus activities of proton-type zeolites without the aid of Ag^+^ and Cu^2+^ generally adopted as active species in antivirus materials (*e.g.*, inactivation of > 99% of M13 phage by 320HOA). TEM analyses confirmed the inactivation of M13 phage and PR8 virus, and the antivirus test in noncontact system suggested that acidic properties on the vicinity of external surface of zeolites would determine the antivirus properties. In this study, we discussed only proton-type zeolites with FAU topology, but as a future perspective, it should be demanded to focus on 3-dimentional topology of zeolites because acidity of zeolites depends also on their topology. Although further studies are still required to reveal the mechanism of antivirus behavior by proton-type zeolites, these findings will open up the possibility to develop low-cost, environmentally and human health harmless antivirus materials.

## Supporting information

S1 Fig Crystal structures of zeolite. Structure models were adapted from IZA database. (TIF)

S2 Fig PXRD patterns of commercially available zeolites with FAU topology. (TIF)

S3 Fig PXRD patterns of as-received Zeoal 4A and ion-exchanged samples. (TIF)

S4 Fig PXRD patterns of as-synthesized EDI zeolite and ion-exchanged samples. (TIF)

S5 Fig Denaturation effect of zeolite samples on RBD domain of spike protein. (TIF)
