# Supplementary figures and images for: Antiviral potential of proton-type zeolite
Source: PLoS One. 2025 May 27;20(5):e0324484. doi: 10.1371/journal.pone.0324484 (PMC12111268; doi:10.1371/journal.pone.0324484)

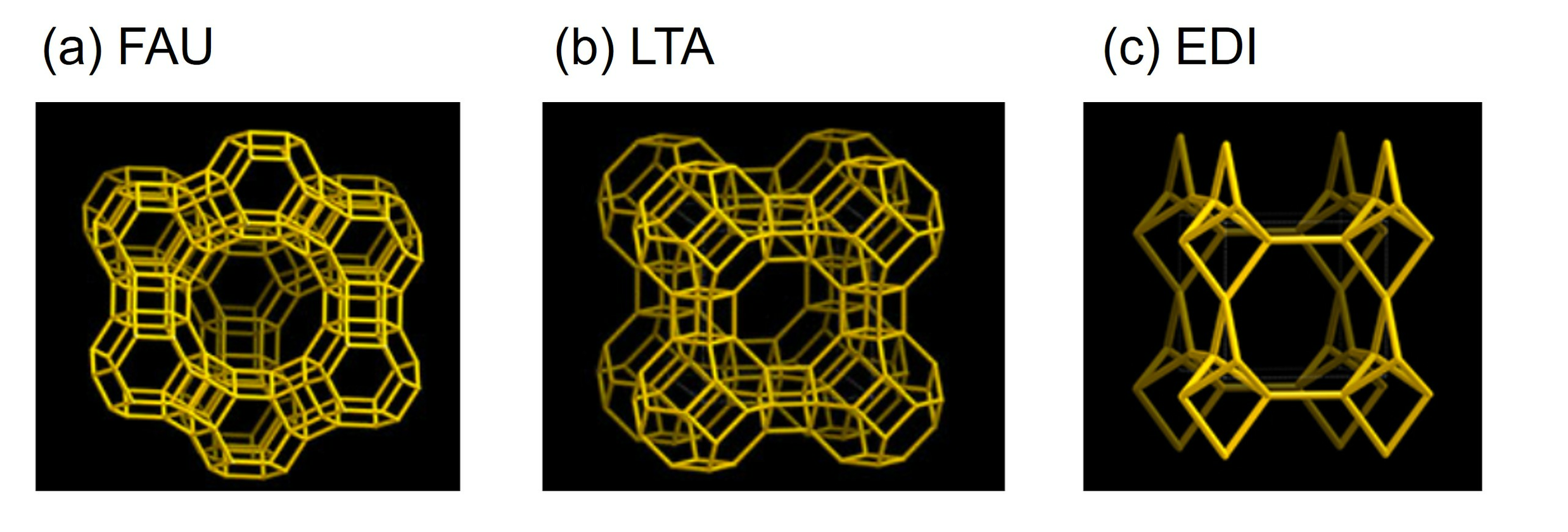

Supplement: S1 Fig — (TIF) [file pone.0324484.s001.tif]

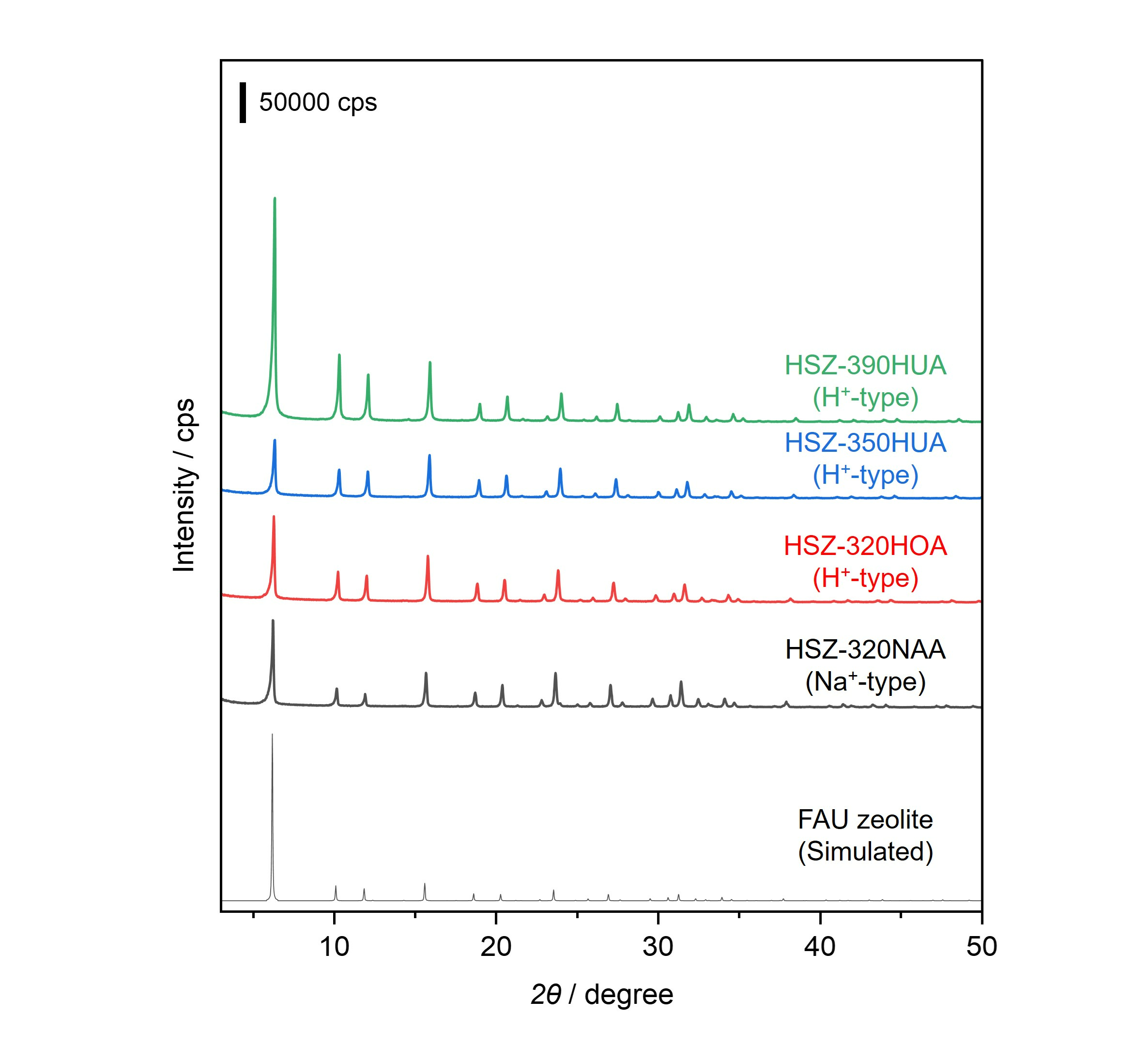

Supplement: S2 Fig — (TIF) [file pone.0324484.s002.tif]

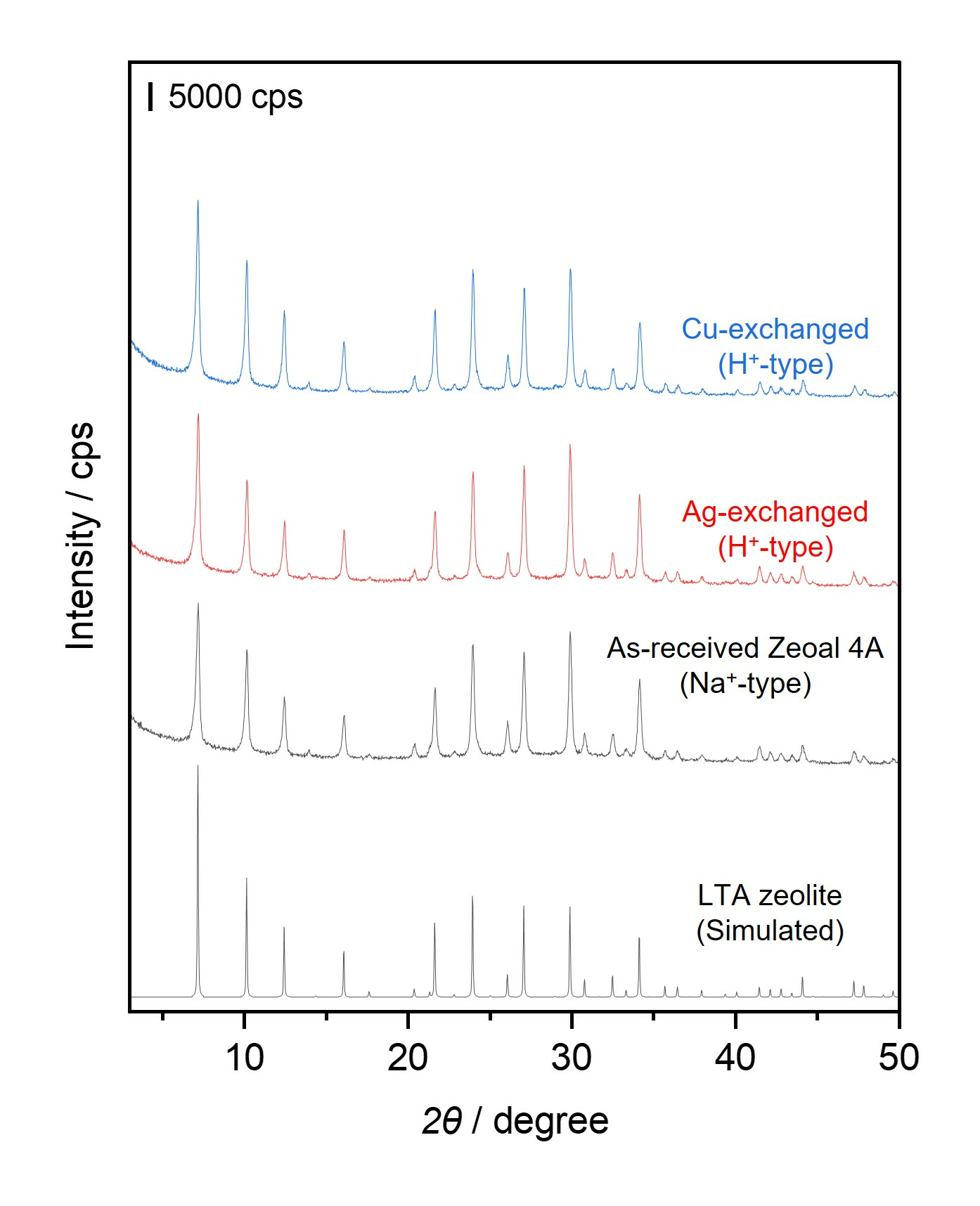

Supplement: S3 Fig — (TIF) [file pone.0324484.s003.tif]

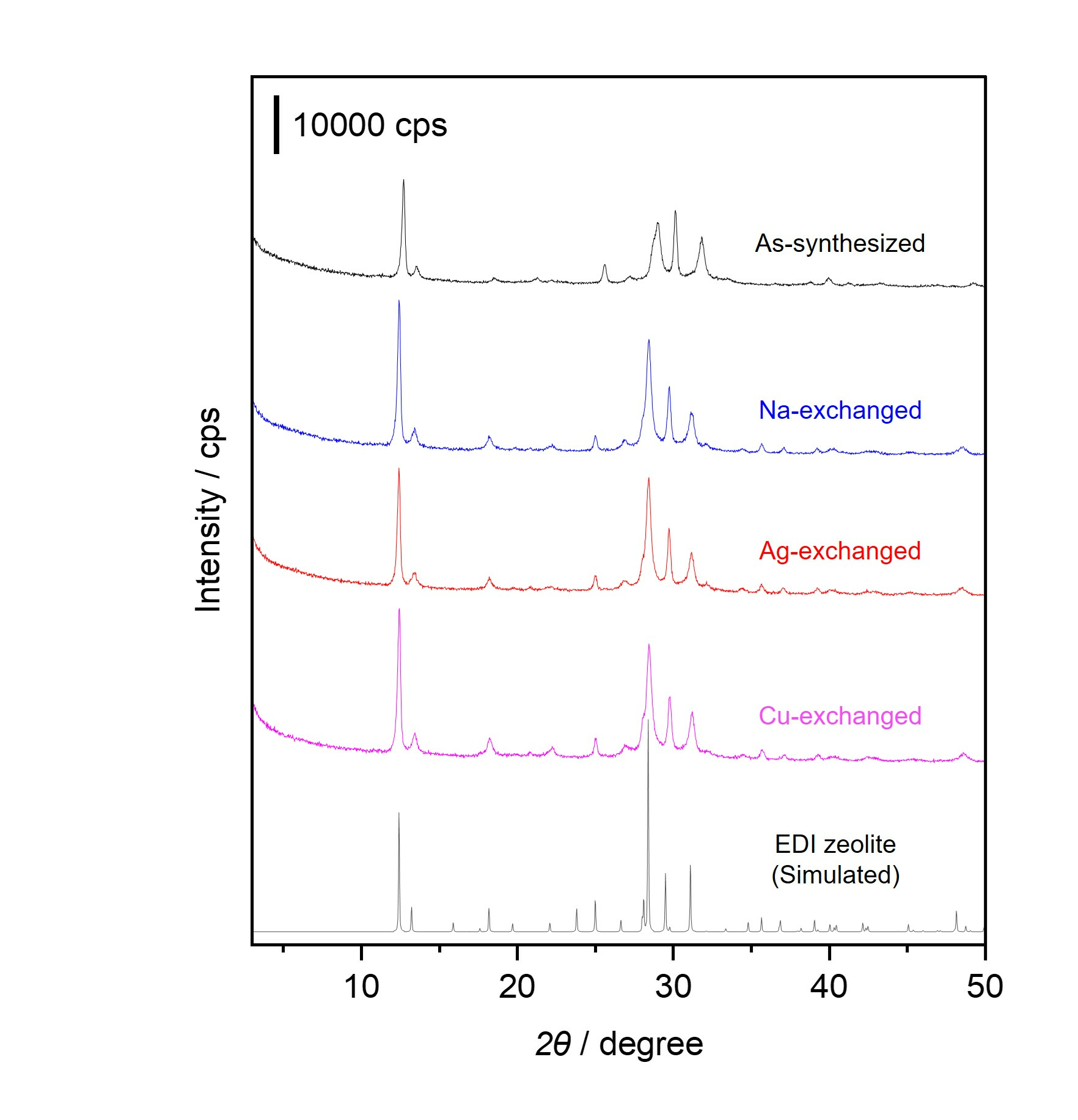

Supplement: S4 Fig — (TIF) [file pone.0324484.s004.tif]

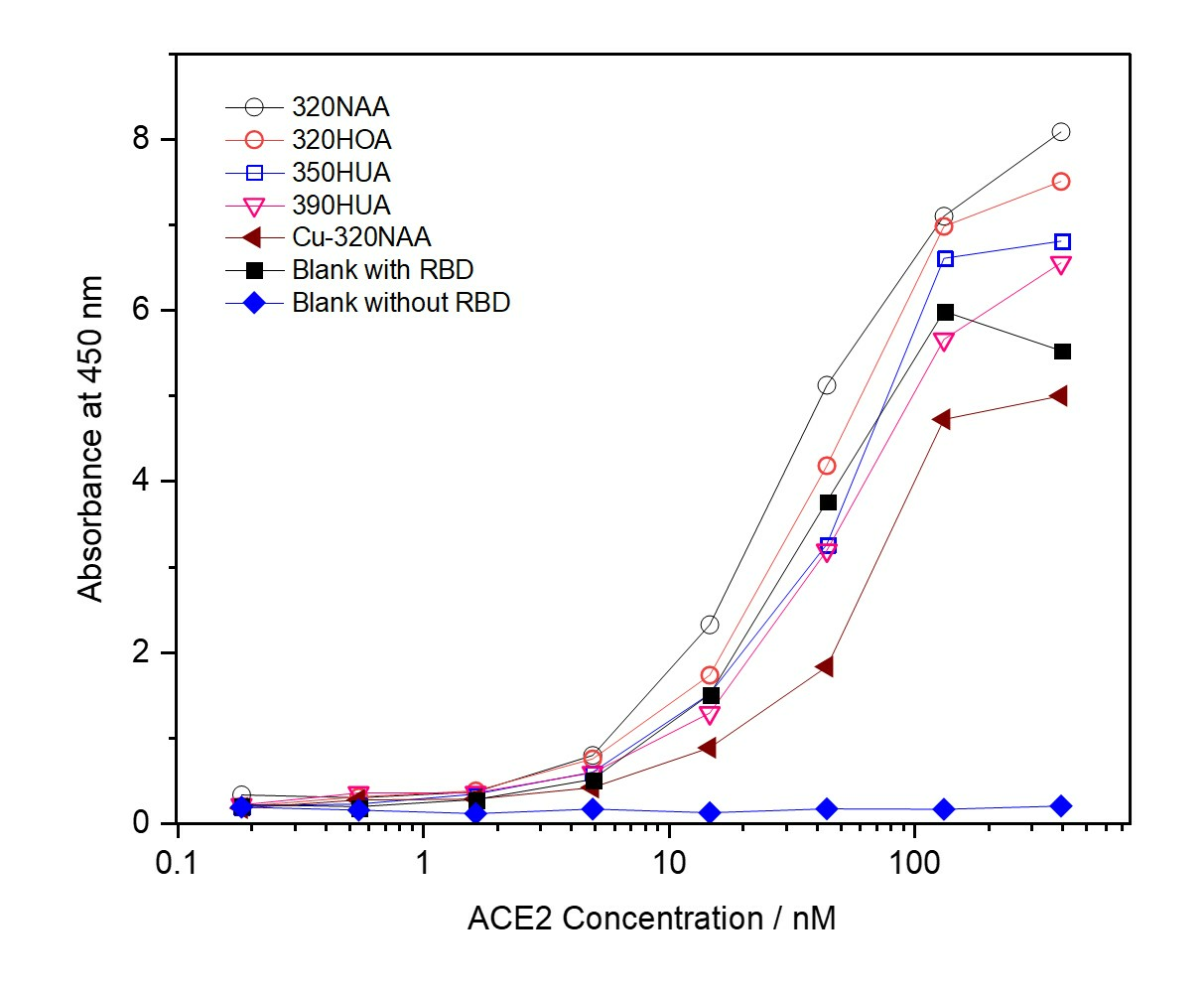

Supplement: S5 Fig — (TIF) [file pone.0324484.s005.tif]
